# Whole Genome Sequencing and Progress Toward Full Inbreeding of the Mouse Collaborative Cross Population

**DOI:** 10.1534/g3.119.400039

**Published:** 2019-03-11

**Authors:** John R. Shorter, Maya L. Najarian, Timothy A. Bell, Matthew Blanchard, Martin T. Ferris, Pablo Hock, Anwica Kashfeen, Kathryn E. Kirchoff, Colton L. Linnertz, J. Sebastian Sigmon, Darla R. Miller, Leonard McMillan, Fernando Pardo-Manuel de Villena

**Affiliations:** * Department of Genetics; † Department of Computer Science, and; ‡ Lineberger Comprehensive Cancer Center, University of North Carolina, Chapel Hill, North Carolina 27599

**Keywords:** whole genome sequence, heterozygosity, Multiparent Populations, MPP, inbreeding, GenPred, Shared data resources, Genomic Prediction

## Abstract

Two key features of recombinant inbred panels are well-characterized genomes and reproducibility. Here we report on the sequenced genomes of six additional Collaborative Cross (CC) strains and on inbreeding progress of 72 CC strains. We have previously reported on the sequences of 69 CC strains that were publicly available, bringing the total of CC strains with whole genome sequence up to 75. The sequencing of these six CC strains updates the efforts toward inbreeding undertaken by the UNC Systems Genetics Core. The timing reflects our competing mandates to release to the public as many CC strains as possible while achieving an acceptable level of inbreeding. The new six strains have a higher than average founder contribution from non-*domesticus* strains than the previously released CC strains. Five of the six strains also have high residual heterozygosity (>14%), which may be related to non-*domesticus* founder contributions. Finally, we report on updated estimates on residual heterozygosity across the entire CC population using a novel, simple and cost effective genotyping platform on three mice from each strain. We observe a reduction in residual heterozygosity across all previously released CC strains. We discuss the optimal use of different genetic resources available for the CC population.

The Collaborative Cross (CC) is a mouse multiparent population (MPP) designed for studying complex traits, modeling human diseases, and is the original stock of the Diversity Outbred population (Iraqi *et al*. 2008; Chesler *et al*. 2008; Morahan *et al*. 2008; Churchill *et al.* 2012; Svenson *et al.* 2012). Over the past several years, the CC has been used to model a wide range of biomedically relevant traits including: allergy (Orgel *et al.* 2018), asthma (Donoghue *et al.* 2017), behavior (Chesler 2014; Schoenrock *et al.* 2018; Molenhuis *et al.* 2018), bone development (Levy *et al.* 2015), cancer (Dorman *et al.* 2016; Reilly 2016), cellular immune phenotypes (Graham *et al.* 2017), drug disposition (Nachshon *et al.* 2016), exercise (McMullan *et al.* 2018), fertility (Shorter *et al.* 2017), glucose tolerance (Abu-Toamih Atamni *et al.* 2017; Nashef *et al.* 2017), infectious disease susceptibility (Durrant *et al.* 2011; Ferris *et al.* 2013; Rasmussen *et al.* 2014; Gralinski *et al.* 2015; Graham *et al.* 2015; Graham *et al.* 2016; Gralinski *et al.* 2017; Green *et al.* 2017; Abu Toamih Atamni *et al*. 2018; Zhang *et al.* 2018; Collin *et al.* 2019), motor performance and body weight (Mao *et al.* 2015), spontaneous colitis (Rogala *et al.* 2014), and toxicology (Cichocki *et al.* 2017; Hartman *et al.* 2017; Mosedale *et al.* 2017; Venkatratnam *et al.* 2017).

The CC is derived from eight inbred strains: five classical strains (A/J, C57BL/6J, 129S1/ SvImJ, NOD/ShiLtJ, and NZO/HILtJ) and three wild-derived strains that represent three *Mus musculus* subspecies (WSB/EiJ from *Mus m. domesticus*, PWK/PhJ from *M. m. musculus*, and CAST/EiJ from *M. m. castaneus*) (Collaborative Cross Consortium 2012). We previously reported on the genome sequence of 69 CC strains (Srivastava *et al.* 2017). We identified a significant reduction in overall genomic contribution from the two strains with non-*domesticus* subspecific origin, CAST/ EiJ and PWK/PhJ. These observations are consistent with the expectation that genetic incompatibilities between the three mouse subspecies contribute to the large-scale extinction of the CC lines during inbreeding (Shorter *et al.* 2017). We also reported on the overrepresentation of the wild-derived strains in regions of residual heterozygosity present within individual CC strains. Residual (*i.e.*, segregating) heterozygosity in the CC refers to regions where more than one founder haplotype at a locus can be found within a single strain.

Publicly available whole genome sequence (WGS) is the new standard for widely used mouse inbred strains (Doran *et al.* 2016; Lilue *et al.* 2018). In MPPs, sequencing helps to resolve recombination breakpoints between consecutive haplotypes, assign the most likely founder haplotype in identical by descent (IBD) regions, and identify strain specific variants (Srivastava *et al.* 2017). However, the sequence of a single individual may provide an incomplete and biased view of an inbred strain, especially when residual heterozygosity remains at relatively high levels. Multiple individuals from a strain need to be analyzed in order to accurately determine the level of residual heterozygosity. The Most Recent Common Ancestors (MRCA/MRCAs) are the best description of residual heterozygosity at the time when a CC strain is released to the public (Welsh *et al.* 2012). In most cases, the MRCAs are the complete set of obligate ancestors. In a few cases, the ancestors were not available and additional individuals were added to better capture patterns of residual heterozygosity. As inbreeding has continued during the maintenance and distribution of the CC, residual heterozygosity is expected to decrease. The time elapsed between the MRCAs and the present should be proportional to the reduction in residual heterozygosity.

Here, we publicly release genetic information (MRCA and whole genome sequence) on six previously unreported CC strains completed by the Systems Genetics Core Facility (SGCF) at the University of North Carolina (UNC). These six genomes have an increased frequency of haplotypes from the two non-*domesticus* inbred wild-derived strains compared to the previously reported 69 strains. The analysis of the inbreeding progress using genotypes from three recent mice from 72 CC strains reveals a decrease in residual heterozygosity across all strains. We discuss the strengths of the available CC genomic resources and plans from the SGCF to track inbreeding progress and residual heterozygosity.

## Materials and Methods

### Mouse Strains

All CC mice were obtained from the SGCF at UNC (Welsh *et al.* 2012). CC mice are publicly available and can be obtained from the SGCF at UNC (http://csbio.unc.edu/CCstatus/index.py) or from The Jackson Laboratory. The MRCAs for the six new strains were born between May 2014 and August 2015. Mice used for sequencing were all males and born between April 2016 and July 2016. Mice used for MiniMUGA genotyping were mostly born between 2017 and 2018. For strains that were extinct or not available at UNC at the time of this study, mice genotyped for MiniMUGA were born between 2015 to 2016 (Table S1).

### Genotyping

All sequenced samples and MRCAs were genotyped using GigaMUGA (Morgan *et al.* 2015). Three additional mice (two females and one male) were selected from 72 CC strains and were genotyped using the MiniMUGA platform (Neogen, Lincoln NE). To ensure the correct mice were sequenced and genotyped on MiniMUGA, each sample’s genotype was compared to the sequenced mouse’s genotype for concordance (Table S2).

### Estimation of residual heterozygosity

To estimate residual heterozygosity in the autosomes and the *X* chromosome in the CC, we used a subset of MiniMUGA markers. These markers are biallelic and informative in the CC founders. The genotypes in the CC founders are consistent among biological replicates and do not contain H or N calls. Finally, genotypes in the founders perfectly predict the genotypes in F1 hybrids. There are 6,293 such markers distributed across the genome. Markers where the three samples per strain had one or more H calls or do not share the same genotype call were treated as evidence of residual heterozygosity. Within each strain, clusters of markers with evidence of residual heterozygosity were combined to conservatively estimate the fraction of the genome with residual heterozygosity. To estimate relatedness between samples genotyped in MiniMUGA and their corresponding CC strain, we used a subset of 3,295 markers that overlapped between GigaMUGA and MiniMUGA genotyping platforms.

### Haplotype reconstruction

Haplotype reconstruction for the MRCAs and each sequenced sample was performed as previously described in Srivastava *et al.* 2017 to identify the founder haplotype contribution across the genome. Founder haplotypes are reported as a probability vector for each of the 36 possible founder states (8 inbred, and 28 founder-pairs) at each marker position.

The obligate ancestors for each line were genotyped using a combination of MegaMUGA and GigaMUGA platforms as described previously (Morgan *et al.* 2015). In the case where an ancestor was genotyped using the more dense GigaMUGA platform, MegaMUGA founder probabilities were imputed from a forward-backward Hidden Markov Model (HMM) that was used to estimate the 36-state probability at each GigaMUGA marker based on its genotype (Srivastava *et al.* 2017). MegaMUGA probabilities were imputed via linear interpolation at MegaMUGA marker positions. For samples genotyped on MegaMUGA a forward-backward HMM was used to directly estimate the founder-state probabilities.

The founder-state probabilities for each strain are estimated by combining the probabilities of the strain’s obligate ancestors using the following rules. At each marker, a maximum probable founder-state was determined for each of the obligate ancestors. If the maximum likelihood was inbred, but from different founder states, then those probabilities were redistributed (added) to all combinations of heterozygous states involved (ex. *p* for AA, was added to the AB probability if BB was the highest probability for the marker in any other obligate ancestor for the strain and the probability of AA was set to 0. Likewise the sample or samples with BB as the maximum probability with value *r*, *r* was added to AB and BB set to 0). Once the discordant inbred probabilities were redistributed within a sample’s marker, the maximum probability for each founder-state was then selected from the set of obligate ancestors and the resulting vector of 36 maximum probabilities were normalized. These normalized probabilities are reported as the strain’s haplotype reconstruction.

There are small differences in how sex chromosomes are treated by the pipeline. For males, the forward-backward HMM has only 8-states for the *X*-chromosome, but when merged with the female obligate ancestors they are extended to 36 with the heterozygous states set to 0 probabilities, and they are then combined into strain probabilities as before.

### Sequencing

Whole genomic DNA was isolated as described in Srivastava *et al.* (2017) from tail tissue of a single male from the following six CC strains: CC078/TauUnc, CC079/TauUnc, CC080/TauUnc, CC081/Unc, CC082/Unc and CC083/Unc. Samples were sequenced at the UNC High Throughput Sequencing Facility (HTSF). DNA was sheared by ultrasonication, and size selection was targeted at 350 bp using a PippinPrep system. The HTSF generated sequencing libraries using Kapa (Kapa Biosystems) DNA Library Preparation Kits for Illumina sequencing. Each CC sample was run in two lanes of HiSeq4000 (Illumina). Paired end 150-bp sequencing was performed on these samples.

### Burrows-Wheeler transforms

FastQC (v0.11.5) was used to confirm the quality of the raw sequencing reads. Multi- string Burrows-Wheeler transforms (msBWTs) were then constructed using the raw fastq sequencing reads of each CC sample. The msBWTs were constructed using ropeBWT (Li 2014) and the msBWT merge algorithm (Holt and McMillan 2014). The msBWT data structure is a lossless compressed representation of the raw sequenced reads that can be efficiently queried for any specific subsequence. The msBWTs were employed in two pipelines. The first was to query each genome against the set of annotated variants from the Sanger Institute (Keane *et al.* 2011) that are informative among the CC founders. This set of queries was described in Srivastava *et al.* 2017. The msBWTs were then queried against 45 base pair subsequences (both forward and reverse complement) from the *Mus musculus* reference genome (GRCm38.68) overlapping by 15 base pairs. The resulting read counts were archived as a data matrix and used for deletion discovery.

### Large deletions

We identified homozygous genomic deletions greater than 500 base pairs in non- repetitive genomic regions that were unique to each strain. This was accomplished using msBWT queries of overlapping 45-mers shifted by 15 base pairs from the GRCm38v68 reference genome. The msBWT reports the number of reads containing each 45-base substring in both forward and reverse-complement orientations. In order to mitigate for random sequencing errors and contamination due to “index-hopping” (van der Valk *et al.* 2018), we selected a minimum threshold of at least 3 reads as sufficient evidence of a 45-mer’s presence in each sample. Once missing 45-mer intervals were identified the msBWT was used to extract the flanking non-zero reads at the interval boundaries, and a consensus sequence was constructed for 45 to 60 bases inside of the missing interval. If this consensus was consistent with a simple polymorphism the new consensus sequence was used to extend the flanking sequence, and the msBWT was used to further extend into the near-zero interval until either the gap closed, or the new consensus’ edit distance from the reference was inconsistent with a simple polymorphism.

### Data availability

Genotypes for MiniMUGA and GigaMUGA are available at the MMRRC-UNC https://www.med.unc.edu/mmrrc/genotypes. The 36 state probabilities and haplotype reconstruction of the MRCAs for the six additional CC strains are publicly available at http://csbio.unc.edu/CCstatus/index.py?run=FounderProbs. Sequenced genomes can be queried using the msBWT tools available at http://www.csbio.unc.edu/CEGSseq/index.py?run=MsbwtTools. The sequenced samples are available at ENA accession PRJEB31495 as FASTQ files. Zenodo accession no. 2586963 provides access to the genotypes from MiniMUGA and GigaMUGA, ideogram and haplotype files based on GigaMUGA genotypes, whole genome sequence, and MRCAs. Supplemental material available at Figshare: https://doi.org/10.25387/g3.7814399.

## Results and Discussion

The six strains reported here were not included in the initial report of the CC genomes (Srivastava *et al.* 2017) because of differences in health status and high levels of residual heterozygosity. Three strains (CC078/TauUnc, CC079/TauUnc and CC080/TauUnc) were quarantined to remove potential pathogens. Five strains (CC078/TauUnc, CC080/TauUnc, CC081/Unc, CC082/Unc and CC083/Unc) had levels of residual heterozygosity over 10%. Typically, <10% residual heterozygosity was recommended before distribution of a CC strain independent of residual heterozygosity levels. While not fully inbred, many CC users will find that these new strains are valuable for genetic mapping, validation experiments, and disease modeling because they add haplotypes from underrepresented CC founder strains.

Based on the MRCAs, the average residual heterozygosity of the six new strains is 19.9%, compared to 8.0% in the previously released strains. Among all 75 CC strains sequenced, five of the new strains are among the seven least inbred strains. In four of these new strains, over 20% of the genome is segregating in the MRCAs (Table 1). CC083/Unc has the unique distinction among CC strains, where three haplotypes are segregating at a locus. Specifically, 129S1/SvImJ, NOD/ShiLtJ and CAST/EiJ haplotypes are present in a 17 Mb region on chromosome *15* (31,869,802-58,735,934). All six new strains have haplotype contributions from all eight founders while almost 20% of the previously released strains have only six or seven founders (Table 2). Overall, three founder haplotypes (A/J, CAST/EiJ, PWK/PhJ) are overrepresented and two (NOD/ShiLtJ, NZO/HlLtJ) are underrepresented in the six new strains (Table 2). Importantly, two of the overrepresented founders (CAST/EiJ, PWK/PhJ) are of non-*domesticus* origin (Table 1 & 2). These two founders were underrepresented in the previous report (Srivastava *et al.* 2017). We also previously reported that wild derived founder haplotypes were significantly overrepresented in regions of residual heterozygosity in the CC (Srivastava *et al.* 2017). We observe the same pattern in the new CC strains where wild-derived haplotypes are enriched in regions of residual heterozygosity (*P* < 0.0001).

**Table 1:** MRCA information for the six new sequenced strains

Strain and mouse ID	% Heterozygous in MRCA	% Heterozygous in sequenced sample	% Heterozygous in 3 miniMUGA genotypes
CC078/TauUnc_M1502	14.29996	1.7	5.52
CC079/TauUnc_M1086	6.766071	4.5	8.62
CC080/TauUnc_M1283	22.18861	3.77	17.11
CC081/Unc_M332	21.91887	3.7	18.81
CC082/Unc_M505	22.76935	5	18.84
CC083/Unc_M3234	31.48258	15.4	28.87

**Table 2:** Founder haplotype frequency in sequenced samples

Population		A/J	C57BL/6J	129S1/SvImJ	NOD/ShiLtJ	NZO/HlLtJ	CAST/EiJ	PWK/PhJ	WSB/EiJ
Released CC strains	Average	0.117	0.150	0.146	0.142	0.137	0.094	0.085	0.128
	Min	0.000	0.000	0.000	0.000	0.000	0.000	0.000	0.000
	Max	0.297	0.338	0.282	0.317	0.374	0.201	0.173	0.354
New CC strains	CC078/TauUnc	0.176	0.166	0.142	0.113	0.046	0.098	0.136	0.123
	CC079/TauUnc	0.199	0.147	0.130	0.086	0.109	0.157	0.135	0.038
	CC080/TauUnc	0.163	0.223	0.151	0.059	0.182	0.055	0.027	0.140
	CC081/Unc	0.089	0.148	0.181	0.154	0.153	0.071	0.101	0.096
	CC082/Unc	0.148	0.101	0.062	0.107	0.150	0.177	0.099	0.149
	CC083/Unc	0.177	0.166	0.142	0.110	0.046	0.098	0.136	0.123
	Average	0.159	0.159	0.135	0.105	0.114	0.109	0.106	0.112

**Figure 1:**
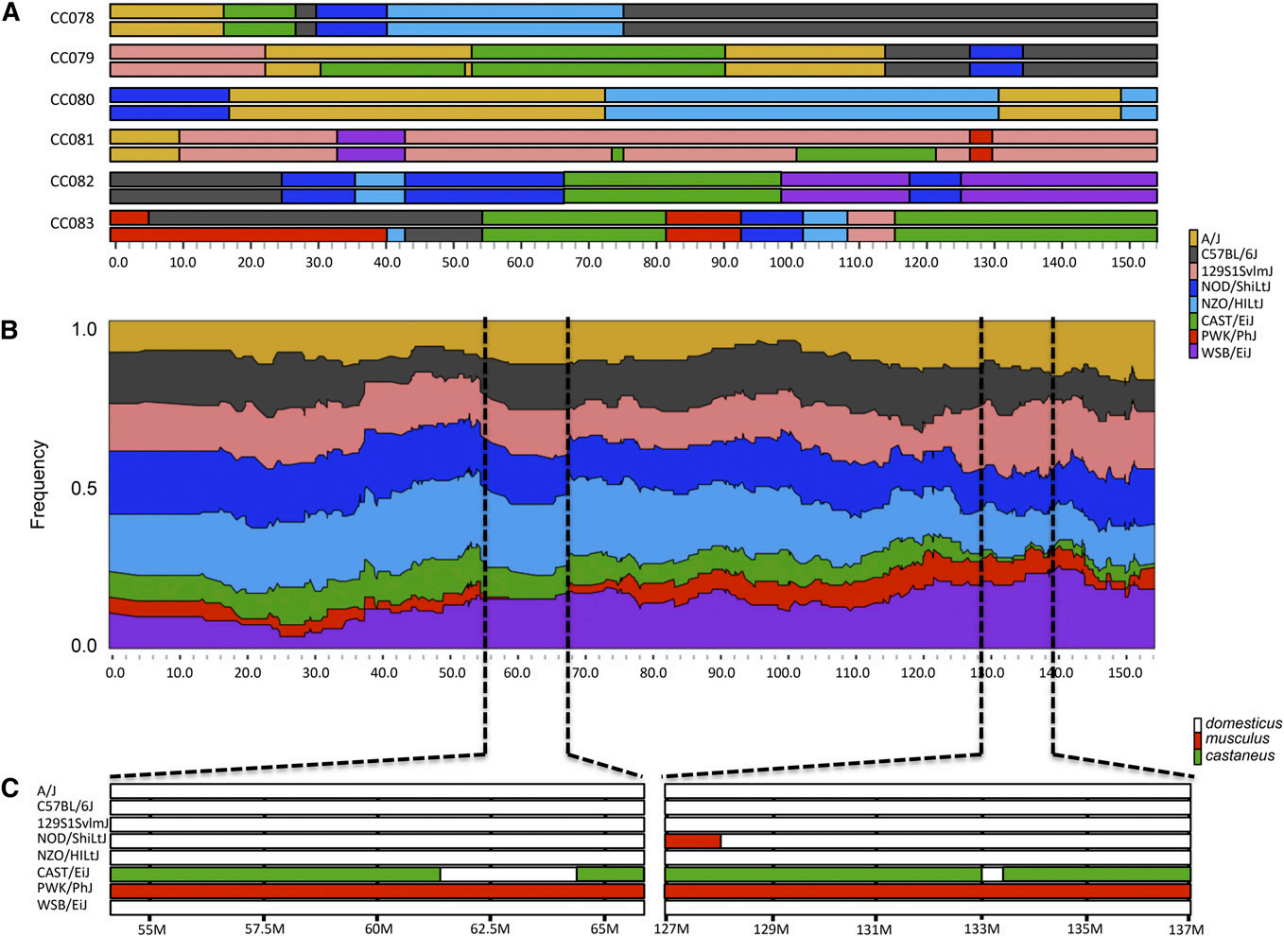
Genomic contribution of new strains to the CC resource on chromosome 5. (A) Haplotype structure for the 6 new sequenced strains. (B) Current haplotype frequency among the previously sequenced 69 strains. Dotted lines represent areas with low PWK or CAST representation. (C) The substrain contribution across the eight CC founders. The CAST founder has genomic regions of *domesticus* origin within the highlighted area. We use the following colors and letter codes to represent the eight founder strains of the CC: A/J, yellow; C57BL/6J, gray; 129S1/SvImJ, pink; NOD/ShiLtJ, dark blue; NZO/HlLtJ, light blue; CAST/EiJ, green; PWK/ PhJ, red; and WSB/EiJ, purple.

The six new CC strains contribute at least one CAST/EiJ or PWK/PhJ haplotype to 62% and 54% of the genome, respectively. This partially relieves the deficit of these underrepresented haplotypes in the CC population. For example, chromosome *5* contains a locus between 127Mb and 137Mb where currently only one CC strain has a CAST/EiJ founder haplotype (Figure 1). The new CC strains add an extra homozygous CAST/EiJ haplotype into the CC population on chromosome *5*. Another example is chromosome *X* where the previously released CC strains have a low frequency (< 5%) of haplotypes from CAST/EiJ or PWK/PhJ. The six new CC strains add at least one haplotype from CAST/EiJ or PWK/PhJ, spanning 70% and 41% of the entire *X* chromosome, respectively. The addition of strains with underrepresented haplotypes is important for different reasons. First, it increases power for genetic mapping in the CC simply by increasing the number of tested haplotypes. Additional strains with the relevant haplotypes also enable independent validation experiments in the CC (Shorter *et al.* 2017). Interestingly, there are regions in the CC population where non-*domesticus* haplotypes are not represented at all (chromosome *5*: 61Mb to 64Mb; Figure 1). These areas have a decreased non-*domesticus* haplotype frequency on the periphery of the highlighted area, suggesting selection is acting against those particular haplotypes in the CC.

**Figure 2:**
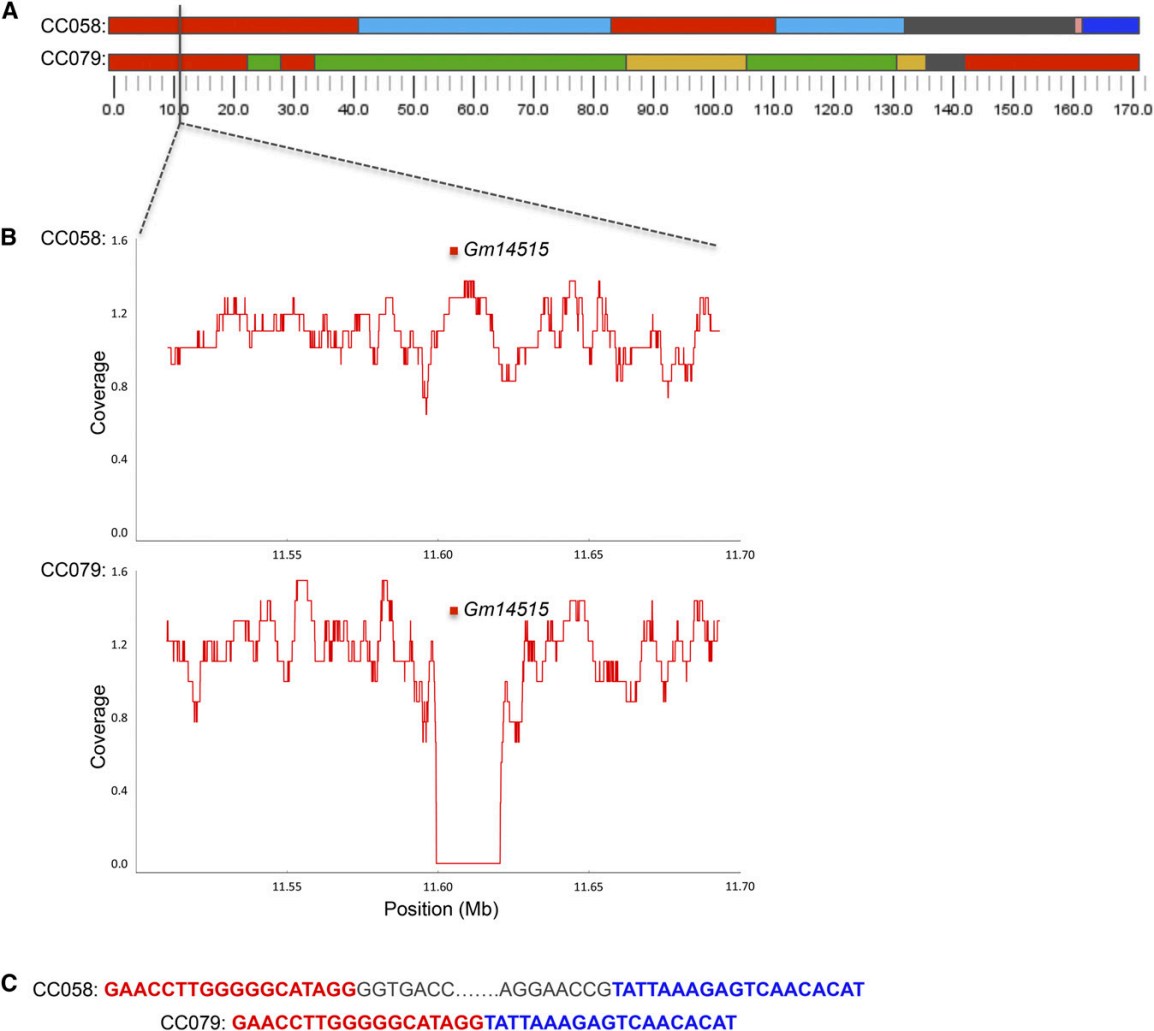
*De novo* private deletions in the new CC sequenced strains. (A) A deletion on the *X* chromosome in CC079/TauUnc not shared with CC058/Unc, which also has a PWK haplotype. (B) The normalized whole-genome coverage of sequence in 1-kb bins for CC079/TauUnc. The deletion spans the pseudogene *Gm14515*. (C) Assembled sequencing from msBWTs shows the breakpoint of the deletion.

The eight founder strains of the CC have been sequenced at high depth, their variants annotated, and their genome recently *de novo* assembled (Keane *et al.* 2011; Doran *et al.* 2016; Lilue *et al.* 2018). Previous genetic variation analyses have used sequence from these founders to infer haplotype specific genetic variation in the CC (Durrant *et al.* 2011; Ferris *et al.* 2013; Gralinski *et al.* 2015; Oreper *et al.* 2017). By directly sequencing CC strains, we can define founder specific variants across multiple CC strains sharing a founder haplotype at each locus, and identify strain specific variation unique to a CC strain. We first compared the sequenced samples’ GigaMUGA genotypes and confirmed that they are consistent with the corresponding MRCA. Next, we queried variation within identical founder haplotype regions at any given locus across the CC to identify *de novo* mutations. We identified four unique *de novo* deletions larger than 500bp in the newly sequenced mice that are not present in the assembled reference genome of the CC founders or in the previous cohort of 69 sequenced CC mice (Table 3). Figure 2 shows a 19.8kb deletion in CC079/TauUnc that is predicted to delete the *Gm14515* gene. The breakpoints were resolved using msBWTs to locally assemble sequence flanking the deletion. A PCR assay was designed to discriminate between the presence or absence of this deletion, and the deletion was fixed in both the MRCA and the three CC079/TauUnc mice tested. We conclude that this mutation arose before or during the early CC inbreeding stages. Mutation and genetic drift may cause distinct variation to arise and become fixed in individual CC strains leading to genetic differences between founder strains and their haplotypes in the CC population.

**Table 3:** Analysis of *de novo* deletions

Strain	Chr	“45-mer” Start	“45-mer” End	Resolved start	Resolved end	Size (kb)	Reads spanning deletion boundaries	Haplotype	Genes	Regulatory Elements	Notes
CC079/TauUnc	*8*	102931561	102947356	102931583	102947323	15.795	na	129S1/ SvlmJ	None	ENSMUSR00000737813	
CC079/TauUnc	*X*	11600371	11620171	11600372	11620112	19.8	11	PWK/ PhJ	*Gm14515*	ENSMUSR00000759295	Independent validation
										ENSMUSR00000759296	
										ENSMUSR00000759294	
CC079/TauUnc	*2*	129264616	129265201	129264705	129265185	0.585	37	PWK/PhJ	*Gm14024*		
CC081/Unc	*10*	56858446	56862406	56858470	56862407	3.96	40	C57BL/6J	None		

**Figure 3:**
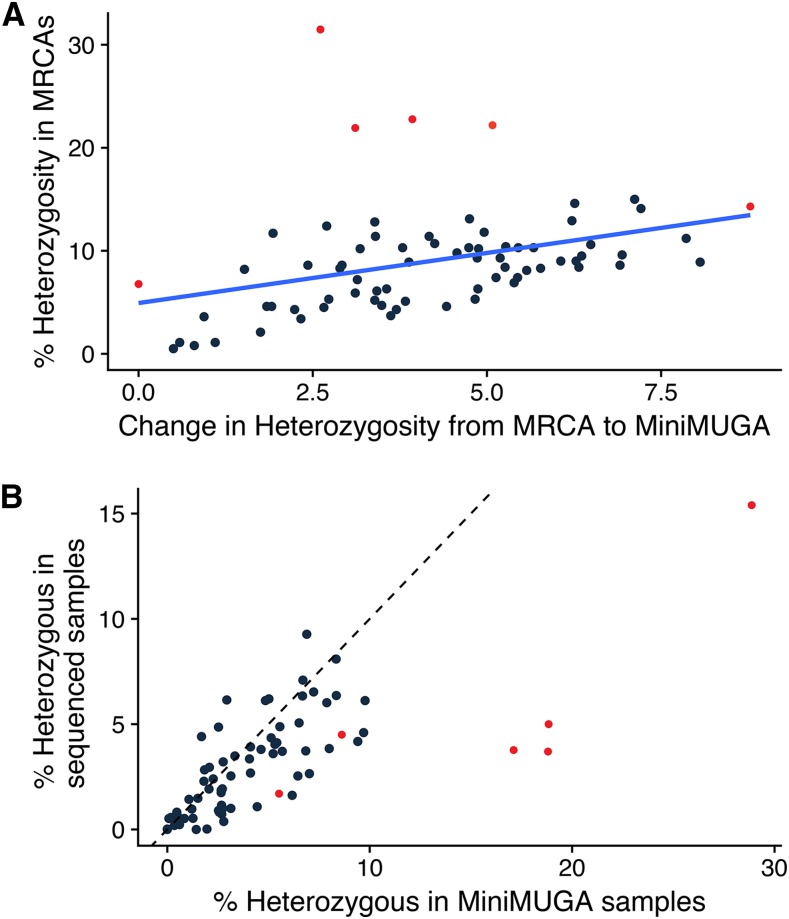
Residual heterozygosity levels in CC populations. (A) Change in CC strain heterozygosity compared to total percent heterozygosity. Red dots represent the 6 new strains. Dark blue line represents the regression for change in heterozygosity compared to heterozygosity levels in the MRCAs. (B) Heterozygosity levels in sequenced samples compared to heterozygosity levels in MiniMUGA samples. Dotted black line represents the equal level of heterozygosity between both populations. Dots to the right of the dotted line are samples where the sequenced sample underestimates heterozygosity and dots to the left are where the sequenced sample over represents heterozygosity.

To better characterize inbreeding progress in the CC, we performed a colony survey using the newly developed MiniMUGA array to genotype three mice (two females and one male) for 72 CC strains, including the six newly sequenced strains. We selected this sex ratio to sample the *Y* chromosome in each colony and to increase the *X* chromosome representation in the analysis. First, we confirmed that all sampled mice had consistent *Y* and mitochondria with their respective MRCA and sequenced sample. Next, we confirmed that all sampled mice had autosomal genotypes that matched with their respective sequenced sample using 3,295 markers common to MiniMUGA and GigaMUGA (Figure S1, Table S2, Table S3). We observe that each of the 216 samples most closely match their respective sequenced sample. This set of markers is sufficient to distinguish two closely related CC strains (CC051/TauUnc and CC059/TauUnc) (Figure S1). The colony survey also allows us to estimate current levels of corresponding MRCA (Figure 3A). The four clear outliers that have both a high level of current heterozygosity and a small decrease in residual heterozygosity are four of the six new CC strains. We hypothesize that the overrepresentation of non-*domesticus* haplotypes combined with high initial residual heterozygosity and small number of generations between MRCAs and genotyped samples contributes to the low change in residual heterozygosity.

The CC population now has three distinct resources that describe their genomes. The first resource is genotype and haplotype reconstructions for the MRCAs of each CC strain. The second resource is whole genome sequence from a single male for 75 CC strains. The third resource is a colony survey of 72 CC strains using MiniMUGA. On average, 2.4 years separate MRCAs from sequenced samples and three years separate sequenced samples from the colony survey. However, there is considerable variation for the lengths of these periods among CC strains (Table S1). These three resources have complementary strengths that can help users of the CC.

The MRCAs, being the most conservative estimate of haplotype composition from the CC colony, are the most robust for genetic mapping, especially when mice were acquired from a wide time range, or sampled from colonies maintained by individual investigators. Users of the CC should be aware that genetic drift has likely fixed regions of residual heterozygosity in the MRCAs, as shown by the MiniMUGA residual heterozygosity analysis (Figure 3A). At regions with residual heterozygosity in the MRCAs, experimental mice could have any of the three combinations of the two segregating haplotypes. However, experimental mice will not contain any additional haplotypes not already in the MRCAs. Mapping power will be reduced in analyses where the MRCAs shows residual heterozygosity that is actually fixed in an experimental population, but one of the two haplotypes will be correctly classified. The MRCAs can be accessed through http://csbio.unc.edu/CCstatus/index.py?run=FounderProbs as genotype probability files for genetic mapping. The probability files are downloadable for each individual MRCA as well as a strain consensus probability file in the GRC mouse build 38.

The whole genome sequence from a single male for 75 CC strains is an excellent resource to define consistent founder haplotype specific variants, identify *de novo* mutations not present in the genomes of the eight CC founder strains, and improve resolution in the transition regions between founder haplotypes. However, attempting to conduct genetic mapping based on the sequenced sample would lead to systemic under-sampling of haplotypes segregating in the SGCF colony. For 51 strains (72% of the sampled CC), the sequenced sample underestimates residual heterozygosity compared to the three more recent samples genotyped in MiniMUGA (Table 1, Figure 3B). On the other hand, identification of potential causative variants within candidate intervals following genetic mapping should rely on the sequenced CC mice relative to the sequenced founder strains. This alleviates potential differences between the sequences of the founders (Keane *et al.* 2011; Doran *et al.* 2016; Lilue *et al.* 2018) and the CC (Srivastava *et al.* 2017), and includes strain specific variants. We created a search tool for comparing sequence across the CC to provide CC users with easy access to sequence data. The msBWT k-mer search is a fast and user-friendly tool to query any k-mer shorter than read length and returns matching sequence http://www.csbio.unc.edu/CEGSseq/index.py?run=MsbwtTools.

The colony survey of 72 CC strains using the set of 6,293 markers defined in the Materials and Methods is a resource that will allow CC users to confirm the strain ID of their CC mice through genotyping. The genotypes of these strains are available at https://www.med.unc.edu/mmrrc/genotypes. Using a subset of 3,295 markers that overlap between MiniMUGA and GigaMUGA, we verified that genotypes of these mice were consistent with the sequenced samples and can be used as a comparison resource (Figure S1, Table S2, Table S3). We recommend that users of the CC preserve tissue samples from all CC mice (and derivatives) used in an experiment. Genetic mapping using genotypes from parental mice (*i.e.*, breeders) would provide the most accurate estimation of current segregating haplotypes for that population. This will also facilitate the correction of potential errors in mouse identity, and serve as a validation of key resources. Our colony sampling of the CC at the SGCF highlights that residual heterozygosity is decreasing, but still present. For experiments where residual heterozygosity could disrupt analysis (*i.e.* vQTL mapping studies, F_2_ mapping studies, recombinant inbred crosses), accurate analyses and interpretation of experimental results will likely benefit from genotyping individual experimental animals to ensure accurate haplotype assignment. Residual heterozygosity may also influence genetic mapping and the presence of QTL, if only one of the two haplotypes at a locus influences a trait (Rogala *et al.* 2014). QTL that have residual heterozygosity may be a double-edged sword. Fixation of a haplotype (or population subsampling) may impede experimental replication in later studies. Alternately, residual heterozygosity can allow for QTL validation studies utilizing a segregating locus within a strain.

Given the level and potential effects of residual heterozygosity in many of the CC strains, regular and comprehensive updates on the heterozygosity still remaining within the SGCF’s colony of CC mice would improve the value of the CC resource. Therefore, the SGCF plans on heterozygosity segregating in the colony over time. Finally, it would be helpful to expand whole genome sequencing beyond a single mouse per strain to ensure that mutations that arise in the colony are taken in to account and potentially exploited in genetic analyses (Kumar *et al.* 2013).
